# Study on the Integration Strategy of Online EOL Testing of Pure Electric Vehicle Power Battery

**DOI:** 10.3390/s23135944

**Published:** 2023-06-26

**Authors:** Huazhang Wang, Hang Qin

**Affiliations:** School of Electrical Engineering, Southwest University for Nationalities, Chengdu 610041, China; qinhang@stu.swun.edu.cn

**Keywords:** power battery, EOL testing, detection system, UDS, human–machine interface

## Abstract

This paper analyzes the electrical test items of the EOL testing line in automotive manufacturers. On this basis, this paper proposes and designs an integrated and automated testing strategy to deal with the problems of slow testing speed, high dependence on manual labor and low efficiency. This article mainly analyzes the various tests of the two main tests in battery EOL testing: Battery Management System (BMS) testing and electrical testing. We propose an innovative integrated solution based on various testing items, including the reception, transmission, and self-analysis of different UDS protocol messages, a unique automated electrical performance measurement scheme, and a requirement and logic design of an integrated software end based on Python. The experimental results of actual testing show that the implementation of the integrated strategy greatly reduces the complexity of the testing steps, improves the testing efficiency, and reduces errors caused by human operation.

## 1. Introduction

The environmental impact brought by global warming in recent years has gradually aroused great attention around the world, and vigorously developing new energy vehicles and controlling greenhouse gas emissions have become the widespread consensus among various countries and regions around the world. The EU launched the “European Green Deal” in 2019 to solemnly commit to the world to reduce emissions targets: that is, to reduce greenhouse gas emissions by 50% by 2030 and strive to reach 55% (based on 1990) to ensure that carbon neutrality is achieved by 2050. Last year, each of the EU countries reached a new energy agreement again: to promote the development of new energy sources and to ban the production of new fuel vehicles from 2035 onwards. The United States, for its part, expects to reach a goal of 50% industry penetration of new energy vehicles by 2030. China has also released the New Energy Vehicle Industry Development Plan (2021–2035), aiming for new energy vehicle sales to reach about 20% of total new vehicle sales by 2025 [1].

Pure electric vehicles are the mainstream models of new energy vehicles, with good market prospects and strong market competitiveness. As batteries are the energy source of new energy vehicles, how to predict the battery’s state accurately under complex working conditions has also become a global challenge that needs to be solved urgently [2,3]. At present, during the production of pure electric vehicles, manufacturers generally conduct EOL (End of Line) diagnostic tests on the power batteries of pure electric vehicles to ensure product performance.

**Figure 1:**
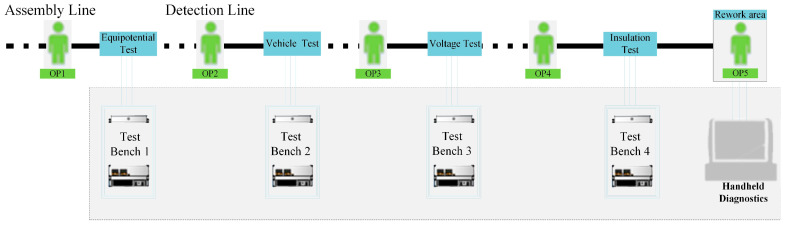
EOL testing process example.

The earliest EOL testing relied entirely on manual labor. With the popularization of general-purpose measuring instruments, EOL testing gradually began to change from relying on manual experience to standard instrumentation, which has greatly improved a range of aspects, from testing efficiency to testing accuracy. However, as the automotive industry evolves, vehicle electronics are becoming increasingly complex. To address this, the International Standard Organization (ISO) has developed corresponding power battery diagnostic standards, including ISO 14229 [4] and ISO 15765 [5]. With the electrical and electronics industry continually improving, it is necessary to conduct an online integrated study of the EOL testing shown in Figure 1. This will improve the testing process while adhering to the international and national standards. Ultimately, this will promote the implementation of relevant standards and improve the stability of pure electric vehicles.

**Figure 2:**
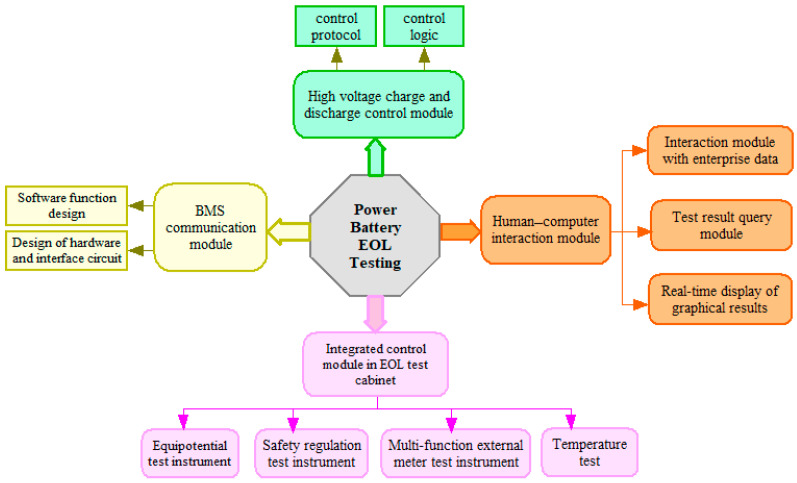
Schematic diagram of EOL system testing structure.

Intelligent testing of new energy vehicle batteries is one of the most important steps to ensure the safety of the entire vehicle. In order to conveniently, quickly and efficiently test the performance of the battery pack, various performance indicators are dynamically displayed in real-time. The entire system includes four main modules: the BMS communication module, the integrated control module in EOL test cabinet, the high voltage charge and discharge control module, and the human–computer interaction module. A schematic diagram of the EOL system testing structure is shown in Figure 2 below:

In summary, the following contributions have been made by this article:

(1) By conducting a detailed investigation of the EOL test, the typical test content of EOL is divided into two categories. The content and qualification standards of the EOL test are sorted out, and the integration strategy of the EOL test is separately studied.
(2) A detailed interpretation of various message mechanisms of the Unified Diagnostic Services (UDS) protocol in BMS testing, including single-frame message, multi-frame message, NRC and DTC, and the possibility of automatic analysis and integration are studied.
(3) The concept of including the electrical performance detection in the integration research is proposed. The working principle and control method of the power relay in the power battery are studied. The specific content and method of electrical performance detection are fully displayed, including some program control methods for the electrical performance detection equipment.
(4) The applicability of LabVIEW and Python in this article is compared and the possibility of using Python language and its rich library resources as an integrated strategy development tool is analyzed.

## 2. EOL Testing Content and Criteria

**Figure 3:**
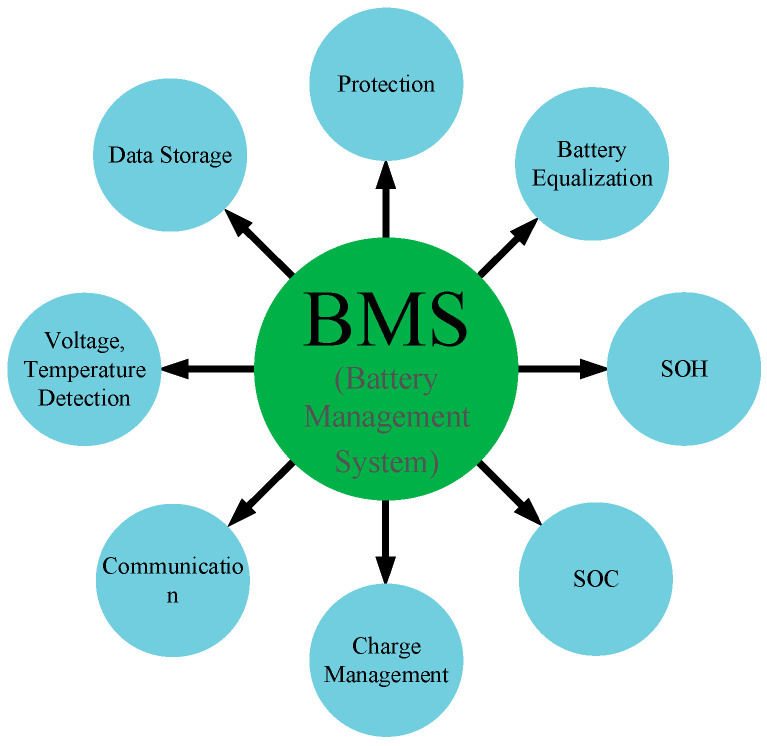
BMS function in a power battery.

The EOL testing for a single battery generally includes SOC (State of Charge), SOH (State of Health) and safety performance. The accurate state parameter, including the core factors such as SOC, SOH, state of power (SOP), and RUL, is the basis for ensuring safety and effective control [6]. However, for power batteries consisting of hundreds or thousands of cells, battery packaging theory and structural integration, management system and methods, and safety control technology are key factors in pure electric vehicle applications [7]. Therefore, EOL testing for power batteries is much more complex. Due to the complexity of the automotive power battery, a BMS (Battery Management System) has been derived, as shown in Figure 3.

**Figure 4:**
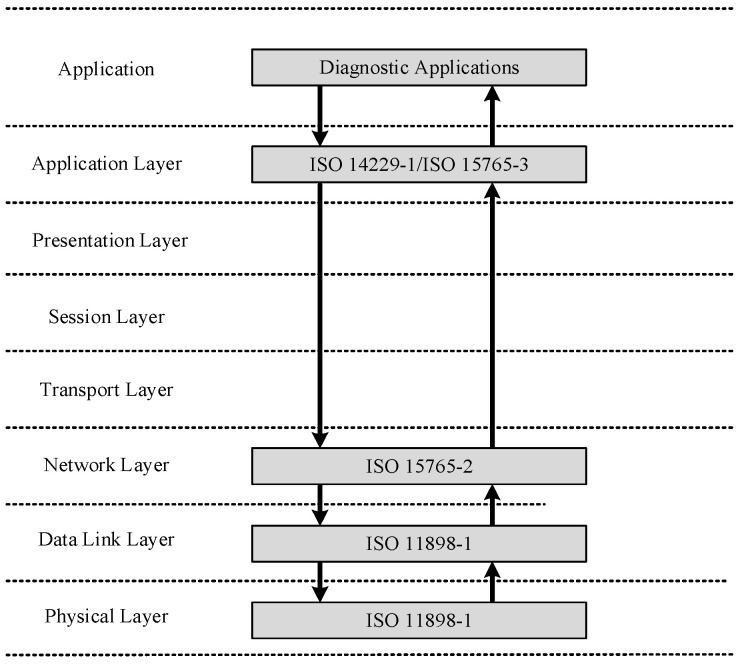
OSI model of the UDS protocol [9].

The BMS reads the sensor parameters integrated inside the power battery to monitor the core parameters inside the automotive power battery in real time, providing information on remaining power, battery status, current, etc. This can prevent over-charging, over-discharging, over-voltage, over-current and an overly high temperature of the battery. The merits and drawbacks of the BMS directly affect the service life of the power battery pack [8]; a suitable battery management system provides the best protection and ensures optimal battery performance, lifespan, and lower operating costs for electric vehicles. Thus, it is essential to test the BMS function in the EOL testing; ISO 14229 and ISO 15765 provide detailed specifications for the communication protocol of the BMS, and Figure 4 illustrates the OSI model of this protocol.

The electrical performance of the power battery is also the focus of EOL testing, and the requirements and testing methods for the electrical performance of automotive power batteries are presented in GB 18384-2020 [10] and GB 38031-2020 [11]. Moreover, the power system of the power battery usually uses relays to control its output voltage and charging state, and the control module composed of various relays is an important safeguard for the electrical safety of the power battery. Therefore, when testing the electrical performance of the power battery, not only the electrical safety performance needs to be tested, but also the relay performance of the power battery needs to be evaluated.

In summary, the typical test contents and passing criteria of power battery EOL testing are shown in Table 1.

## 3. BMS Detection

The main function of the BMS is to protect the battery [12] and prevent various safety accidents caused by battery failures. The BMS is crucial for the vast majority of electric vehicles [13], so BMS testing must be performed when the power battery goes offline. Pure electric vehicle manufacturers typically manually send measurement instructions for BMS testing. They then analyze the received instructions and calculate measurement results [14]. The contents of the BMS test items in Table 1 can be obtained through the BMS in the power battery, so the main problem for the BMS test is to solve the problem of communication with the BMS and to realize the automatic sending and receiving of BMS data and parsing.

### 3.1. BMS Data Reading Based on UDS Protocol

**Figure 5:**
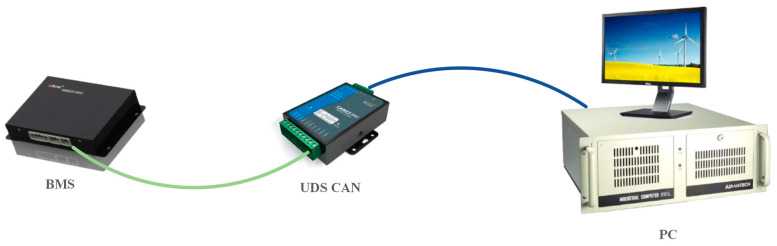
UDS protocol sending and receiving schematic.

ISO 14229 proposes a unified solution UDS protocol (Unified Diagnostic Services) for multiple control units inside the power battery, and ISO 15765 defines how the TP network transport layer handles single and multiple frames for different CAN message formats in the diagnostic model. CAN-based UDS is the preferred solution for BMS testing because the test requires only one test tool compatible with ISO 14229 and ISO 15765 [15,16]. Therefore, the test method used in the actual test process is as shown in Figure 5.

**Figure 6:**
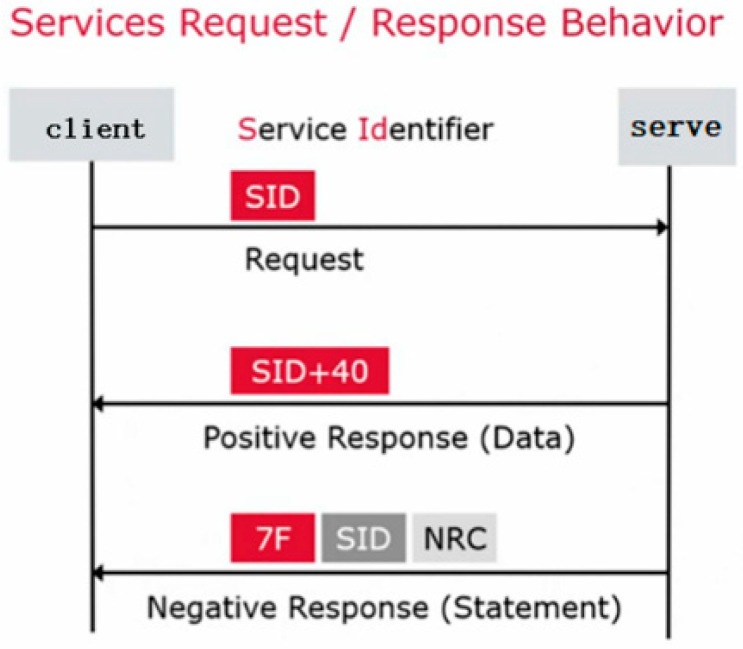
Data transmission rules of UDS protocol.

The UDS protocol specifies the specific way for external devices to communicate with the car battery BMS and uses one byte to represent the diagnostic service, which is called Service ID, or SID for short. The response format is “SID + 40 + specific data”; the negative response format is a fixed format “7F + SID + one byte of NRC in the request message”. When the test does not involve special functions, simply read the corresponding test SID in the SID list to send and receive data according to the fixed target address, and identify the response status of the test by judging whether the SID bits in the received data are equal to the “SID + 40” of the sent data. If the response is positive, the test results of the test item can be obtained by processing the data content of the other data bits in a specified format, as shown in Figure 6.

**Figure 7:**
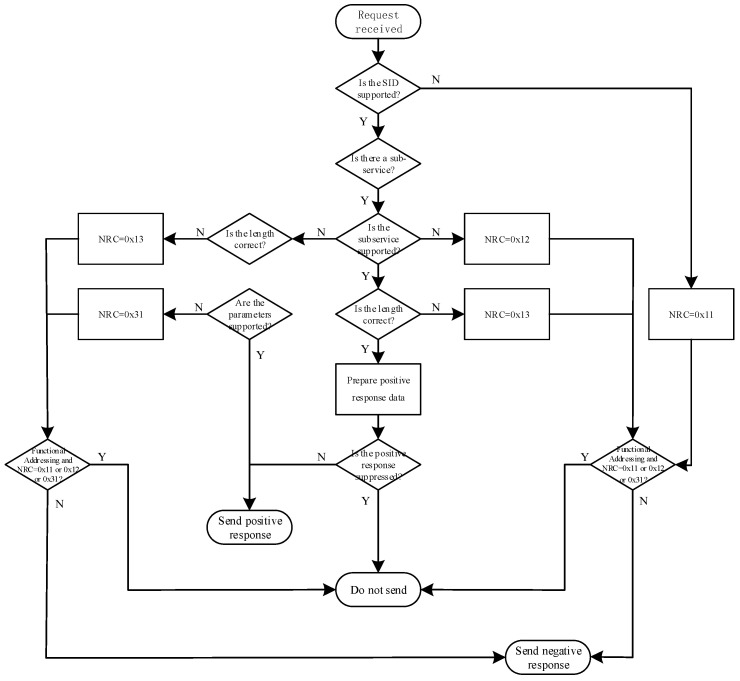
A set of BMS test data.

The NRC (Negative Response Code) is specified in Schedule A of ISO 15765, so when the diagnostic equipment receives the NRC, it is necessary to parse the NRC feedback from the BMS to record the cause and corresponding SID of the generated NRC. However, not all requests will receive the NRC; as a diagnostic device, it can communicate with all BMSs together or specify a BMS alone. Therefore, both Functionally Addressed and Physically Addressed are specified in the UDS. Functional Addressed broadcasts a diagnostic request and waits for the BMS on the bus to respond. Physical Addressed sends a specific diagnostic request and waits for a response from the specified BMS. In the case of physical addressing, the negative response should be sent in the specified format. In the case of functional addressing, when the NRC is 0 × 11 (service not supported), 0 × 12 (subfunction not supported) and 0 × 31 (request out of range), the functional addressing will not send the response. The BMS response rules are shown in Figure 7. Once the corresponding rules are designed, the automatic detection and analysis of regular BMS test items is achievable.

**Figure 8:**
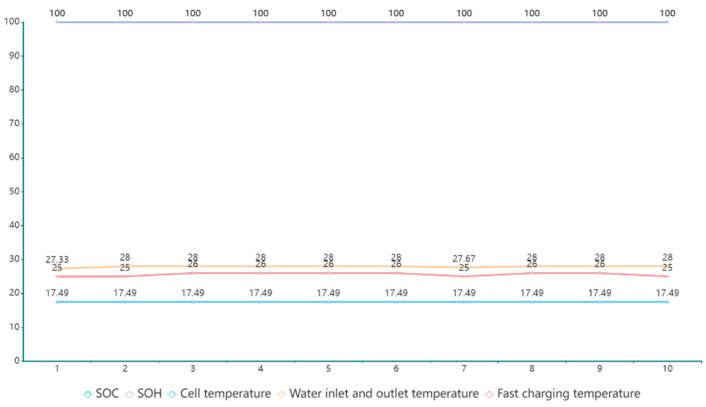
BMS message response mechanism A set of BMS test data.

Figure 8 shows a set of test data for BMS; it includes parameters such as SOC, SOH and some battery temperature parameters. Additionally, the measurement results for each test item in the figure are all qualified. This set of data shows that data obtained from a qualified battery BMS are still relatively stable, which is beneficial for the formulation and implementation of power battery testing standards.

### 3.2. BMS Fault Code Reading and Processing

**Figure 9:**
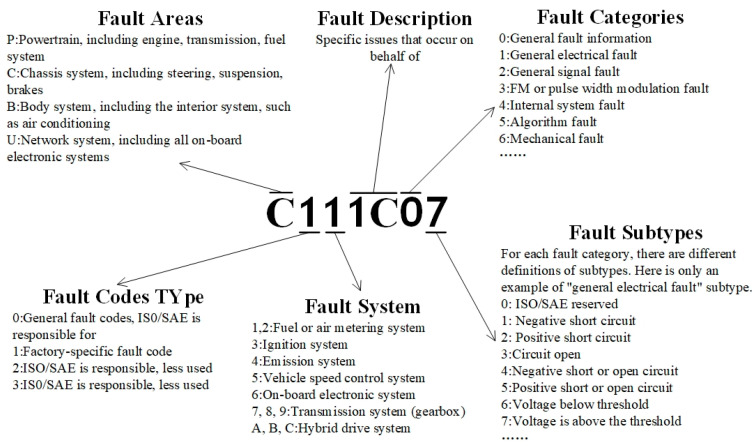
Explanation of the DTC composition.

Trouble diagnosis is one of the important functions of the BMS. The DTC (Diagnostic Trouble Code) is used in the BMS to record the detailed information of vehicle failure, and will alert the user and help prevent accidents. The composition of a trouble code is illustrated in Figure 9 below. In the vehicle EOL testing, the DTC requirement for the power battery BMS is that certain specific DTCs will not appear in different off-line sessions, so the most important thing for the DTC detection in the BMS test is how to read the DTC list in the BMS.

**Figure 10:**
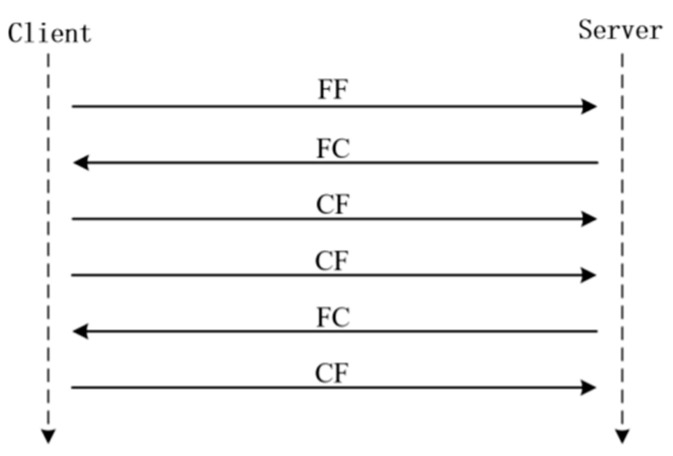
UDS protocol multi-frame data example.

**Figure 11:**
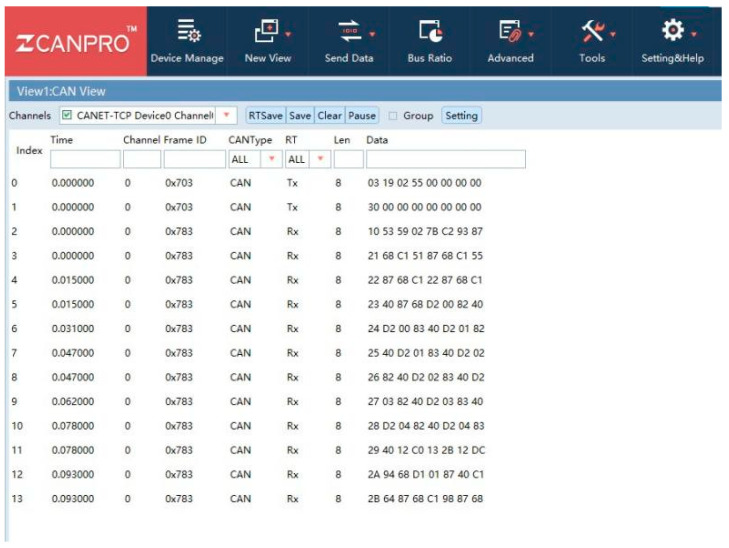
Example of actual measurement of multi-frame data reading.

In the previous subsection, we showed how to read single frame (SF) data from the BMS, but for reading multi-frame data like the DTC list, detailed provisions are also made in the UDS protocol. The multi-frame message generally starts by sending the first frame (FF) to the Server side; after the Server side confirms that the first frame is sent successfully, it sends the flow control frame (FC) to maintain the transmission state. After the Server side receives the flow control frame, it will set the timer to maintain the state according to the STmin (minimum interval of adjacent consecutive frames) in the flow control frame and wait for the request of the Client side to send consecutive frames (CF); when the Server side receives the consecutive frames within the time specified in STmin, it will write them to the buffer and read them. Figure 10 and Figure 11 show an example of multi-frame data reading. The DTC array can be obtained from the BMS through multi-frame reading, and the complete DTC list can be obtained by splitting the array with the specified data format.

## 4. Electrical Performance Testing

**Figure 12:**
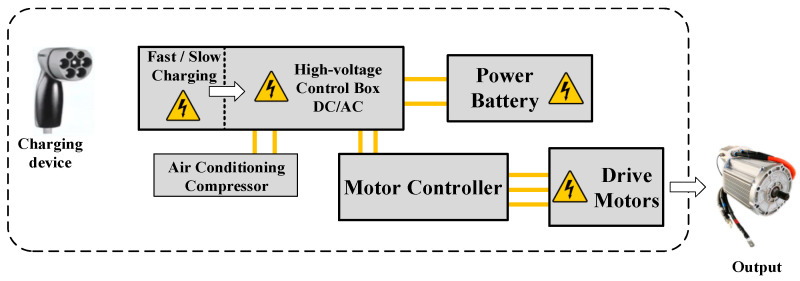
Electrical systems for electric vehicles.

GB 18384-2020 specifies the voltage range of Class B power batteries as 60–1500 V, but in practice, as the power source of pure electric vehicles, the voltage of power batteries is commonly above 300 V [17]. The high-voltage electrical system of pure electric vehicles is defined in the standard GB 39086-2020 as the high-voltage drive component system connected to the DC bus of the power battery or driven by the power battery power source above the B-class voltage inside the electric vehicle, mainly including but not limited to: the power battery system and high-voltage power distribution system (high-voltage relays, fuses, resistors, main switches, etc.), the motor and its control system, the DC/DC voltage converter and on-board charger, the DC voltage converter and on-board charger, etc. [18]. The structure of the high-voltage electrical system of an electric vehicle is shown in Figure 12. It is easy to see that the power battery is the core of the whole high-voltage electrical system, and its electrical performance is crucial for reliability.

As demonstrated in the analysis of the electrical test items of the power battery in Table 1, it is easy to find that the first three test items are for the general testing of electrical performance, while the last three are for the testing of the electrical safety performance of the power battery.

### 4.1. General Electrical Performance Testing

#### 4.1.1. Relay Function Detection of Power Battery

**Figure 13:**
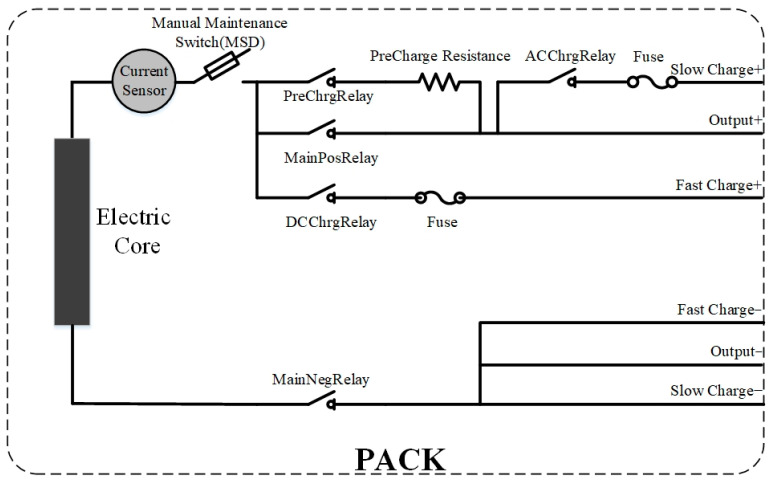
Electrical relationship of power battery relays.

There are generally five relays in the pure electric vehicle power battery: main positive relay (MainPosRelay), main negative relay (MainNegRelay), pre-charge relay (PreChrgRelay), fast charge relay (DCChrgRelay) and slow charge relay (ACChrgRelay). The electrical relationship of the power relays in the power battery is shown in Figure 13. The output combinations of several relays realize different electrical functions of the power battery and also ensure the safety of the power battery to a certain extent.

**Figure 14:**
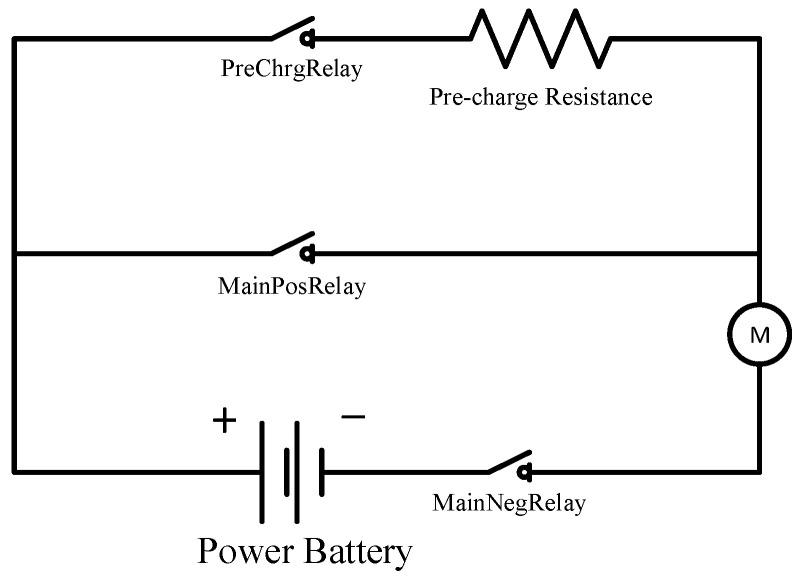
Equivalent circuit for PreChrgRelay operation.

After understanding the electrical relationships of the five relays mentioned above, it can be found that the PreChrgRelay is a relatively special relay in terms of function. Its equivalent circuit with the MainPosRelay is shown in Figure 14. The existence of the PreChrgRelay effectively protects the battery, the MainPosRelay and the MainNegRelay, because when the capacitor is connected in parallel at both ends of the power supply, the moment the power is turned on, the voltage across the capacitor will not change suddenly, but its current will change suddenly. At this time the load resistance is the resistance of the wire and the relay contact, which is generally much less than 20 mΩ, and the battery voltage is generally above 300 V. This is equivalent to an instantaneous short circuit, resulting in an instantaneous current I = 300/0.02 = 15,000 A; the MainPosRelay and the MainNegRelay are easily damaged by overcurrent and overheating.

**Figure 15:**
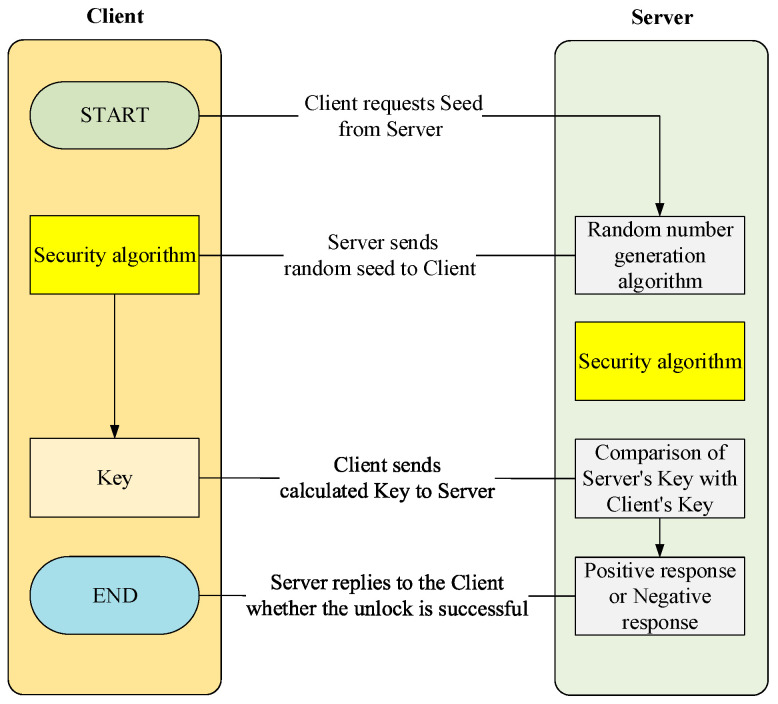
Security algorithm flowchart.

Relays for power batteries are high-voltage relays, for which there are often more stringent control policies to ensure the security of their use. The higher security control privileges required by the 27 service are provided in the UDS protocol, and the different security levels of the service can be defined by each vehicle manufacturer. It stipulates that when certain operations with higher security levels need to be implemented, further security access levels need to be obtained before they can be performed. The acquisition of the security access service seed for external diagnostic equipment and the resolution of the corresponding key are the focus and difficulty of the service, and the specific program control implementation process is shown in Figure 15. Once security access to the 27 service is obtained, the power battery relays can be controlled.

**Figure 16:**
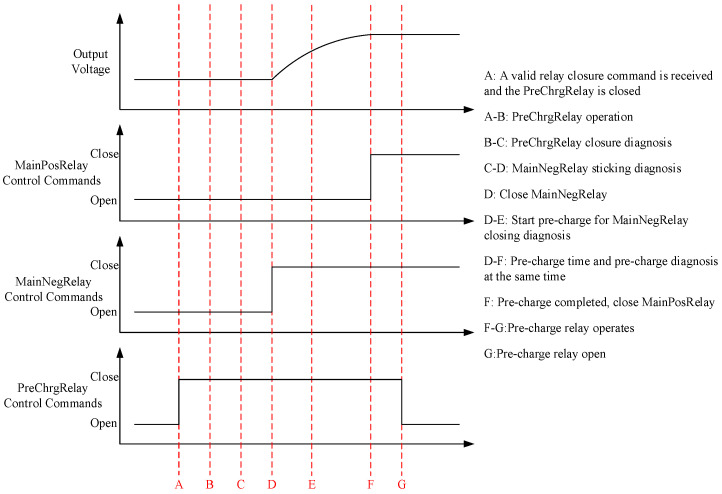
Relay timing in power-up mode.

**Figure 17:**
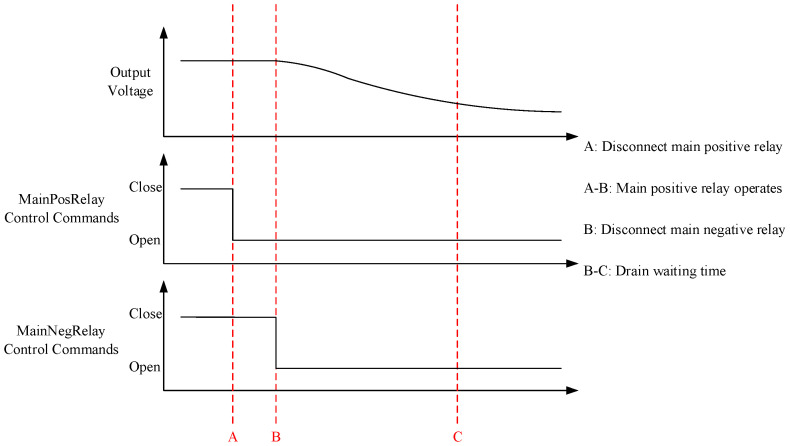
Relay timing in power-down mode.

The electrical functions of the power battery are realized by different combinations of battery high-voltage relays, which mainly include four common electrical functions: power-on mode, power-off mode, slow-charge mode and fast-charge mode. The relay function detection of the power battery can be completed by monitoring the relay timing of the power battery in different modes and measuring the battery output voltage in different modes through the BMS. Figure 16 and Figure 17 are a set of relay timing diagrams measured in the EOL testing process in the up and down modes. By using the program to capture and analyze the key nodes of the timing diagrams and processing the voltage data measured by the measuring instrument, we can determine the good functions of several relays in the diagrams.

#### 4.1.2. Power Battery Voltage Measurement

**Figure 18:**
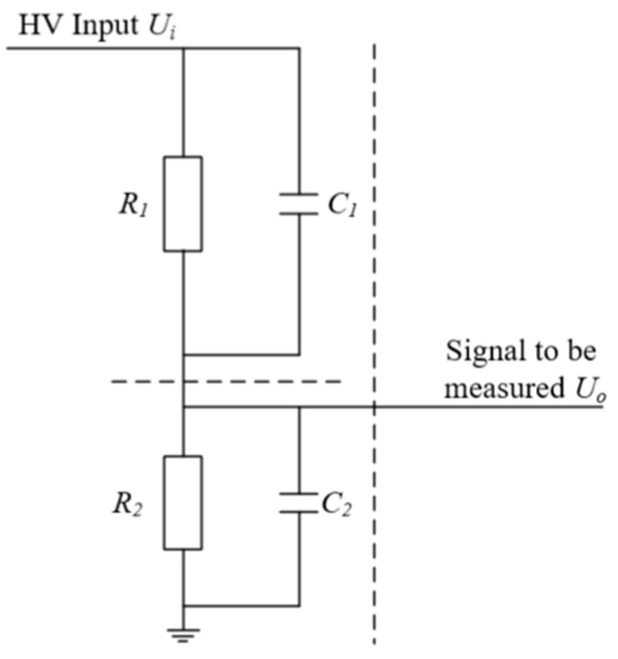
Voltage divider method to measure high voltages.

The voltage of the power battery is a high voltage, although the voltage level of the power battery is not high in the voltage level of the power grid; therefore, many manufacturers for power battery voltage measurement often directly use a desktop digital voltmeter to measure. However, this measurement method often has a high risk and low efficiency, because the high voltage from the terminal is released to the human body; a safe voltage level takes a certain amount of time, for the integration of a challenge, and often one has to set up multiple levels of protection because of the presence of high voltage, which will greatly increase the cost of this type of integrated measurement equipment. There are three DC high-voltage measurement methods commonly used in the power grid: the voltage division method, the high-voltage electrostatic voltmeter, and the ball gap method [19,20]. Table 2 shows the analysis and comparison of these three methods. Through the understanding of these three measurement methods, the voltage of the power battery is innovatively proposed to be measured by the voltage division method in our integration strategy; the measurement principle is shown in Figure 18. After dividing the high voltage *U_i_* at the input in the voltage divider, *U_i_* can be calculated by simply measuring the voltage *U_o_* at the output according to the formula shown in Equation (1).

The equivalent resistance of *R*_1_ and *C*_1_ can be calculated from the above figure:

(1) $$R_{R_{1}C_{1}}=\frac{R_{1}}{1+R_{1}jwC_{1}}$$

The equivalent resistance of *R*_2_ and *C*_2_ is:

(2) $$R_{R_{2}C_{2}}=\frac{R_{2}}{1+R_{2}jwC_{2}}$$

Using the voltage division formula for series resistors:

(3) $$U_{o}=\frac{R_{R_{2}C_{2}}}{R_{R_{1}C_{1}}+R_{R_{2}C_{2}}}U_{i}$$

The Substituting Equations (1) and (2) into Equation (3) yields:

(4) $$\frac{U_{o}}{U_{i}}=\frac{R_{2}}{R_{2}+R_{1}\frac{1+R_{2}jwC_{2}}{1+R_{1}jwC_{1}}}=\frac{C_{1}}{C_{1}+C_{2}\frac{1+1/R_{2}jwC_{2}}{1+1/R_{1}jwC_{1}}}$$

**Figure 19:**
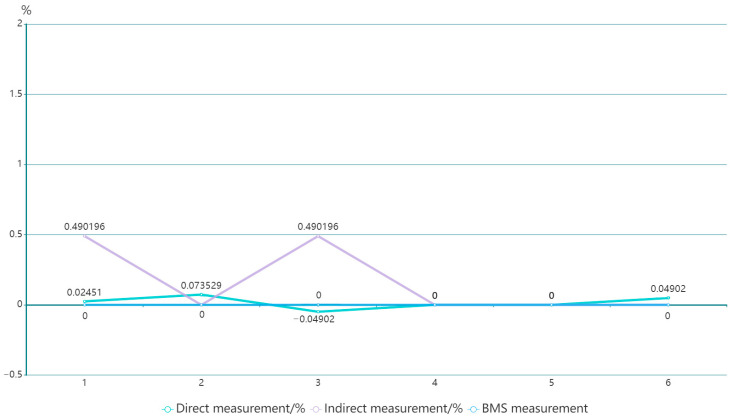
Relative deviation of voltage measurement results.

The high-voltage measurement equipment based on the voltage division method often has a larger size. This is because the measurement upper limit of this type of equipment is usually between several tens to several hundreds of kilovolts. Therefore, the voltage division resistor *R*_1_ of this type of equipment not only requires a larger resistance value but also needs a higher voltage withstand level, which leads to the larger size of the voltage divider. However, the power battery voltage measured in this system usually only has about 400 V, so the voltage division resistor *R*_1_ with a lower voltage withstand level and resistance value can be used, greatly reducing the volume of the voltage divider. In addition, by encapsulating the actual circuit and using high-voltage sealing glue to make a high-voltage package, the high-voltage creeping distance can be further reduced, the volume of the voltage divider can be reduced, and the cost of the integrated device can be reduced. By using this method, only a current protection is needed at the front of the circuit, and the high voltage relay used to measure different high voltages inside the integrated device can be replaced by a low voltage relay, further reducing the cost of the integrated device and ensuring safety. Table 3 shows the comparison of the data of the improved high-voltage measurement device with the direct measurement scheme. Figure 19 calculates the relative deviations of the six measurements from the different measurement methods. The data analysis from the table and the graph indicates that although a small amount of measurement accuracy is lost in the indirect measurement method, a large improvement is achieved in all aspects of measurement safety and control of measurement costs.

### 4.2. Electrical Safety Performance Testing

Any electrical products must undergo mandatory electrical safety testing, and this is also true for automotive power batteries. In 2019, for new energy vehicle battery safety, China formulated and published “GB 18384-2020 electric vehicle safety requirements”, “GB 38031-2020 electric vehicle with power battery safety requirements” and other mandatory national standards [21]; at the same time, the European Union issued ECE R100 and ECE R10, and stipulated that new energy vehicles using power batteries need to compulsorily meet the above documents’ content requirements [22]. Subsequent North American UL 2580, India ALS 038 and other relevant standards [23] have been developed in their respective countries and regions on the electrical safety requirements of new energy vehicle power batteries. Most of the current electrical safety testing relies on testers to use general electrical measuring instruments for item-by-item testing; safety testing of the electrical level will generally be 1.5–3 times the object under test, so such testing still has a high safety risk.

#### 4.2.1. Electrical Safety Testing Content

Three general electrical safety tests are listed in Table 1: the withstand voltage test, potential equalization test, and insulation performance test.

The withstand voltage test is mainly used to apply a voltage higher than the normal working voltage between the output end of the tested power battery and the battery case for a specified period of time; if the leakage current value is less than the standard leakage current threshold of 1 mA, it can be judged to be qualified. The test voltage must gradually rise from zero to the required voltage value within a certain period of time. The purpose of this test is to prove that the material can work safely at the rated voltage or due to switching, arcing and other similar phenomena caused by the instantaneous overvoltage.

When there is no insulation fault, the high voltage wire harness of the power battery is isolated by an insulating layer, and the insulation internal resistance value is in the order of megabytes. If the insulation fault occurs, the insulation performance will be reduced, resulting in safety hazards. Therefore, it is necessary to carry out insulation fault detection. The occurrence of insulation fault means the decrease of insulation internal resistance, and how to obtain the accurate insulation resistance is the key point of insulation fault diagnosis [24]. As per GB 18384-2020 in the electric vehicle power battery, the insulation resistance minimum value is 100 Ω/V; this provision is based on the power battery output and any point between the ground short circuit. The worst-case leakage current will not exceed 2 mA; that is, no harm to humans. In the previous passage, active detection, passive detection, and estimation algorithms were mentioned for testing the insulation performance. The national standard recommends using the pressure difference method for direct measurement; that is, the use of two identical voltage detection tools (known voltage detection tool internal resistance r). While detecting positive-to-shell, negative-to-shell voltage, the reading is stable, and the higher is $U_{1}$, the lower is $U_{1}^{\prime }$; then, the 1 MΩ resistor $R_{0}$ parallel connection in $U_{1}$ side terminal and the shell between the two voltmeters simultaneously detect the positive-to-shell, negative-to-shell voltage. After the reading is stable, the higher is $U_{2}$ and the lower one is $U_{2}^{\prime }$ insulation resistance $R_{i}$. The calculation formula is shown in Equation (5). Although such a measurement method can be a more accurate determination of the insulation resistance of the power battery, the method is more dependent on the testers. In current industrial measurement, the insulation resistance value between the positive and negative terminals of the power battery and the shell (ground) can be directly measured through the measurement procedure set by the integrated withstand voltage tester, which is safer and more reliable.

(5) $$R_{i}=\frac{1}{\frac{1}{R_{0}(\frac{u_{2}^{\prime }}{U_{2}}-\frac{u_{1}^{\prime }}{U_{1}})}-\frac{1}{r}}$$

Requirements for potential equalization testing include:

(a) The connection impedance between the exposed conductive part and the electric platform should be no more than 0.1 Ω;

(b) For a potential equalization pathway, the distance is not more than 2.5 m between the two conductive parts of the resistance and should not be greater than 0.2 Ω.

The significance of the above requirements is that when there is a base insulation failure and the human body is touching any exposed conductive parts, given that the potential balance is small enough, the potential difference can be negligible and the human body will not be electrocuted.

The following Table 4 is a set of data obtained from our actual testing. To comprehensively analyze the test results, a relative deviation is used as the evaluation basis, as shown in Equation (6):

(6) $$R_{d}=\frac{d}{X}\times 100\%$$

**Figure 20:**
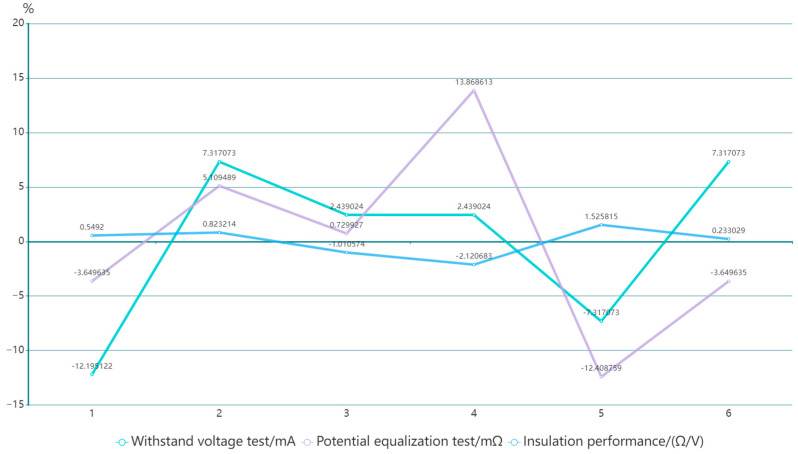
Relative deviation of electrical safety testing results.

In this equation, *X* represents the average value of all test data, and d represents the absolute deviation of the test result, with *d* equal to *x_i_-X*, where *x_i_* is the result at the *i* time trial. Figure 20 shows the absolute deviation of this set of data. Observation of these data results indicates that although there are some deviations in these measurement data, all test data are qualified and far exceed the qualified standards specified in national standards.

#### 4.2.2. Remote Control of Safety Testing Equipment

The development of power electronics has promoted the popularity of universal measurement instruments, which has improved many test operations with high risk factors in the test field. During the rapid rise of the pure electric vehicle field, some general-purpose measuring instruments were gradually involved in the production testing of pure electric vehicles, and the application of these general-purpose measuring instruments in the EOL testing of vehicles is shown in Figure 1.

**Figure 21:**
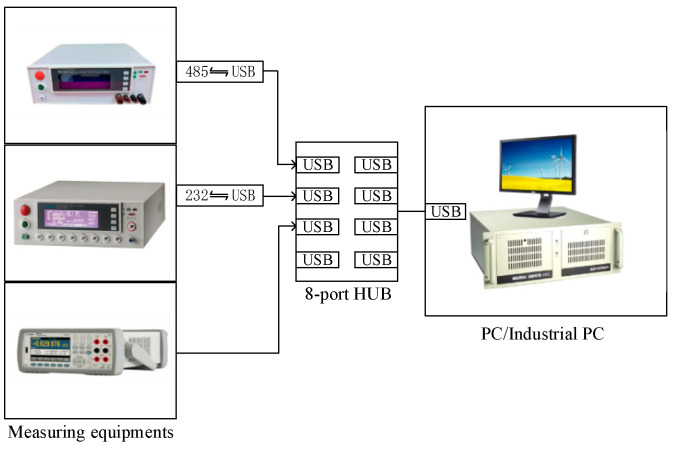
Connection of multi−devices.

**Figure 22:**
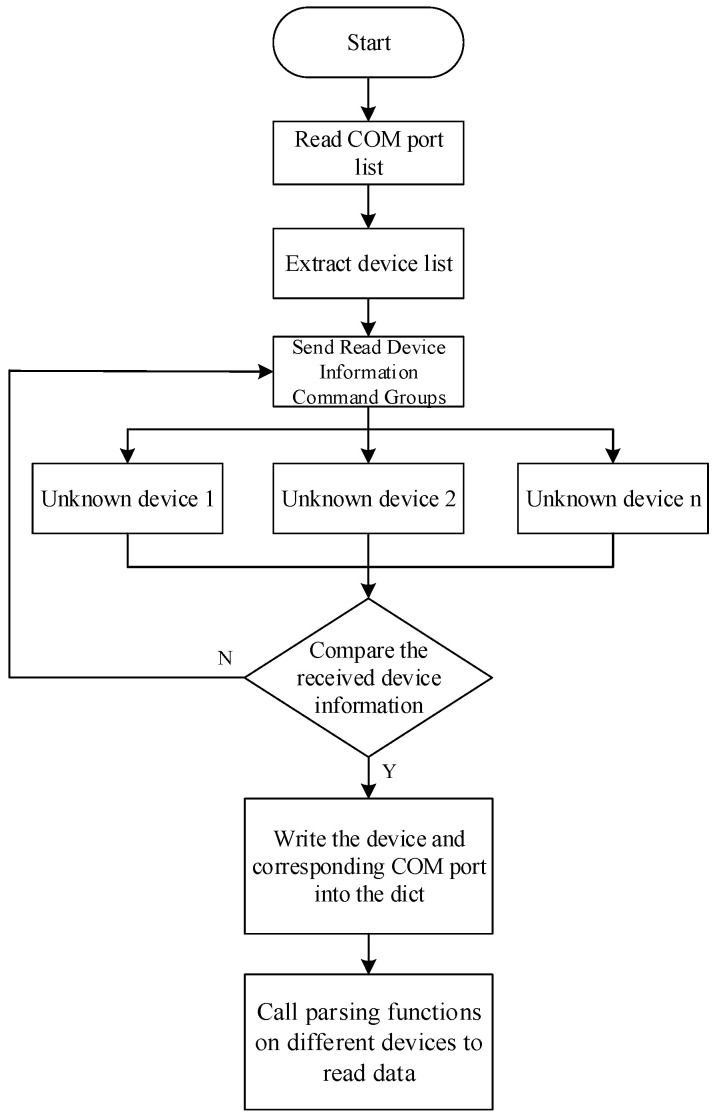
PC-based recognition solution for multiple measurement devices.

In the above, EOL testing items were used with several general measurement instruments, such as the digital multimeter, insulation withstand voltage tester and equipotential tester; in the actual system we choose, the measurement accuracy is higher than the measurement standard measuring instruments to ensure the accuracy of the test. Their remote control used SCPI (Standard Commands for Programmable Instruments) commands. SCPI are a kind of existing standards based on IEEE488.1 and IEEE488.2, and follow the IEEE754 standard in the floating point rules, ISO646 information exchange 7-bit encoding symbols (equivalent to ASCII programming) and other standards of standardized instrument programming language [25]. The EOL testing integration process must solve the problem of remote control of various types of general-purpose measurement instruments; although various general-purpose measurement instrument manufacturers have provided their own equipment remote control program, the collaboration of multiple general-purpose measurement instruments is still a challenge faced in the integration study. At present, the reference approach is directed communication between the embedded system and the controlled instruments [26,27], but this approach often requires a customized embedded system designed according to the actual function and its scalability is poor. After analyzing the information of such devices, we used a multi-devices control approach as shown in Figure 21. By using a conversion port and a hub (HUB), these devices can be easily connected to the same IPC; the COM side of the IPC is scanned, the SCPI’s device information reading command is sent, and the keywords in the intercepted reply message can identify each device of the general measurement information. The control logic is shown in Figure 22.

By using the innovative communication method described above, each general measurement device connected to the computer can be quickly identified during software testing, and its corresponding script can be called to control and read measurement data. This eliminates the need for a customized embedded system. To address the issue of multiple devices alternatingly connecting to the battery output port, simply add a communication-enabled embedded relay system.

## 5. Detection Software Design

**Figure 23:**
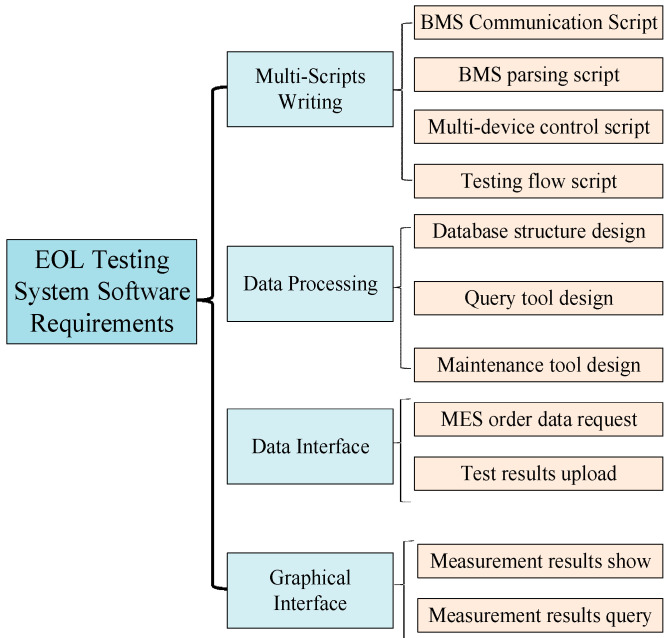
EOL testing software requirements.

The testing software is the most important part of the whole EOL integrated system, and its functional requirements are shown in Figure 23. The testing software not only needs to write scripts for the testing process according to the testing process of the aforementioned measurement items, but also needs to perform operations such as confidentiality, storage, calling and docking of data, etc. In addition, a simple and clear testing interface should be designed for operators to display real-time test results and improve production efficiency.

Currently, most of the production of collaborative software in the industry that involves an image manipulation interface is developed using LabVIEW language, a graphical programming language built specifically for engineering applications in test, measurement or control. However, with the rapid development of Python in the field of automated testing, many complex development processes can be simplified by Python’s rich library resources. Python’s rich resources and excellent performance in data processing and database system are also suitable for the collection, processing and storage of a large amount of test data during EOL testing, so we innovatively use Python and its rich extension resources to develop an EOL testing software.

**Figure 24:**
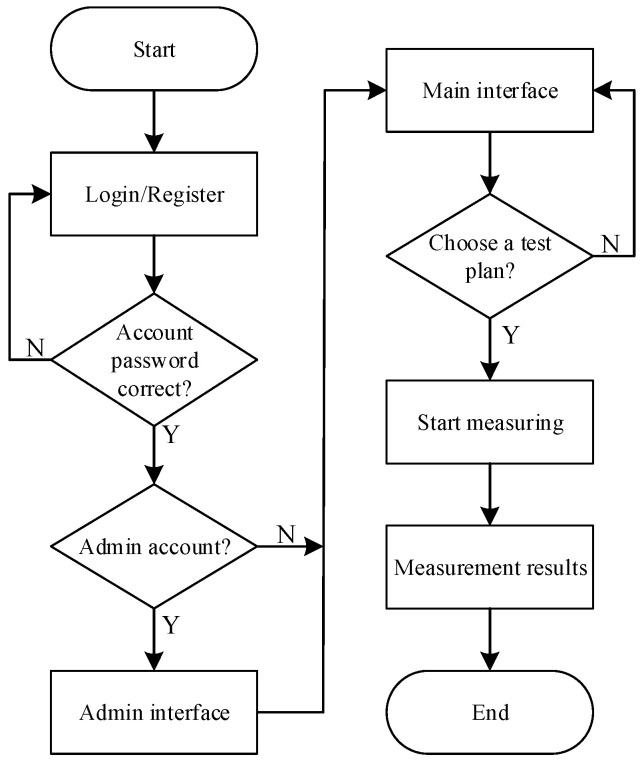
PC software test flow chart.

In the actual research and development process, the PyVisa library in Python can be used to control SCPI devices more conveniently, while the PyQT5 library in Python can make it easier to design a fully functional graphical user interface. This is because PyQt5 successfully combines the Python language and the Qt library, making it compatible with all major operating systems. Due to the special nature of EOL testing, the display interface must be able to show the measurement results of each item in real time. Based on this limitation, a multi-threaded development mode was used in the software design stage; i.e., the main thread only receives and displays the test results, while the child threads make multi-device script calls and process the measurement data, which improves CPU utilization and program execution efficiency. The execution flow of the EOL testing software we wrote is illustrated in Figure 24 below.

**Figure 25:**
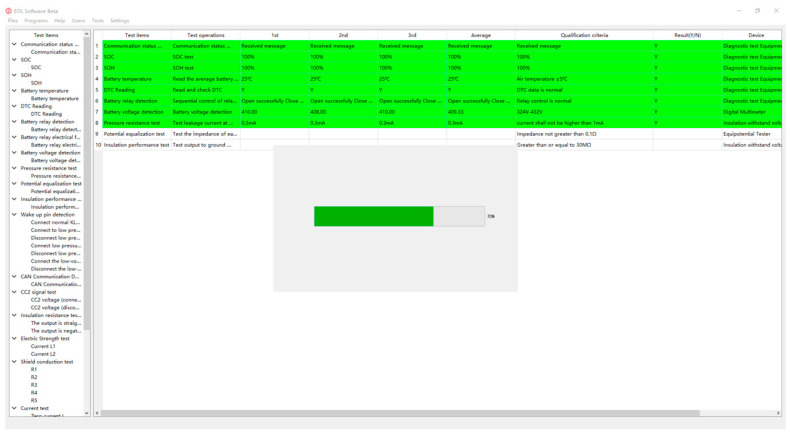
Software actual test example diagram.

Considering the measurement pressure of the testers, we simplified the factors expressed in the test process, and the main interface of the software selected the test items, test content, three tests and test average value, test results and measurement equipment to display the test items. Figure 25 is an example diagram of an actual test. In the actual test, the color hints in the interface can distinguish the measured items and measurement results briefly and clearly, and the testers can select the data display of the interface according to the actual needs to improve the measurement efficiency.

## 6. Conclusions

In this paper, we analyze the test requirements of the current EOL testing process, and reduce the professionalism of the testers in BMS testing by writing directed UDS automation analysis scripts for the test items; through the actual use of each measurement item and each measurement device in electrical testing, we use universal instrument control scripts and unique identification methods to connect all measurement devices to the same online PC. By using a Python solution, which is different from the traditional software solution, the measurement system can be built quickly by making full use of existing resources, and the scripted programming method is conducive to the expansion of measurement items afterwards. In summary, this paper presents an integrated strategy for power battery EOL testing that differs from existing manual measurement approaches. Through research on actual EOL test items, the strategy innovatively achieves automated data analysis of BMS, automated measurement of electrical performance, and an integrated software end based on Python, thereby increasing the safety and automation level of EOL testing while ensuring measurement accuracy.

## Figures and Tables

**Table 1:** Power battery EOL testing content.

Test Type	Test Items	Testing Equipment	Qualification Criteria
BMS testing	Communication status detection	Diagnostic test equipment	Received message
	SOC		100%
	SOH		100%
	Battery temperature		Air temperature ±5 °C
	DTC Reading		DTC data is normal
Electrical performance testing	Battery relay detection	Diagnostic test equipment	Relay control is normal
	Battery relay electrical function testing	Diagnostic test equipment	All electrical status of the battery is normal
	Battery voltage detection	Digital Multimeter	60–1500 V
	Withstand voltage test	Insulation withstand voltage tester	Leakage current shall not be higher than 1 mA
	Potential equalization test	Equipotential Tester	Impedance not greater than 0.1 Ω
	Insulation performance test	Insulation withstand voltage tester	Greater than or equal to 100 Ω/V

**Table 2:** Comparison of three common high-voltage measurement methods.

High-Voltage Measurement Method	Limitations
Partial pressure method	Requires current limitation in the mA class
High-Voltage Static Voltmeter	Small measurement range and high cost for high voltage
Ball gap method	Low accuracy, susceptible to interference, large size

**Table 3:** Voltages measured by the three measurement methods.

Serial Number	Direct Measurement Voltage/V	Indirect Measurement Voltage/V	Voltage Read by BMS
1	408.1	410.0	408.0
2	408.3	408.0	408.0
3	407.8	410.0	408.0
4	408.0	408.0	408.0
5	408.0	408.0	408.0
6	408.2	408.0	408.0
Average	408.1	408.7	408.0

**Table 4:** Electrical safety testing data.

Serial Number	Withstand Voltage Test/mA	Potential Equalization Test/mΩ	Insulation Performance Test/(Ω/V)
1	0.18	2.2	14,311
2	0.22	2.4	14,350
3	0.211	2.3	14,089
4	0.21	2.6	13,931
5	0.19	2	14,450
6	0.22	2.2	14,266
Average	0.205	2.28	14,232.83

## Data Availability

No new data were created or analyzed in this study. Data sharing is not applicable to this article.

## References

[1] Hao H. (2020). Design and Key Performance Study of High-Current Connectors for Electric Vehicles. Master’s Thesis.

[2] Li K., Lu W., Liang C. (2020). Review and prospect of Chinese new energy vehicle power battery from management perspective. Sci. Technol. Manag. Res..

[3] Wang S., Takyi-Aninakwa P., Jin S., Yu C., Fernandez C., Stroe D.I. (2022). An improved feedforward-long short-term memory modeling method for the whole-life-cycle state of charge prediction of lithium-ion batteries considering current-voltage-temperature variation. Energy.

[4] (2013). Road Vehicles Unified Diagnostic Services (UDS) Specifications and Requirements.

[5] (2004). Road Vehicles Diagnostics on Controller Area Networks.

[6] Wang S., Fan Y., Jin S., Takyi-Aninakwa P., Fernandez C. (2023). Improved anti-noise adaptive long short-term memory neural network modeling for the robust remaining useful life prediction of lithium-ion batteries. Rel. Eng. Syst. Saf..

[7] He H., Sun F., Wang Z., Lin C., Zhang C., Xiong R., Deng J., Zhu X., Xie P., Zhang S. (2022). China’s Battery Electric Vehicles Lead the World: Achievements in Technology System Architecture and Technological Breakthroughs. Green Energy Intell. Transp..

[8] Yong F., Hui W., Xiao L. (2010). Research and design of battery management system for pure electric vehicles. Meas. Control Technol..

[9] (2020). Road Vehicles Controller Area Network (CAN)-Part 1: Data Link Layer and Physical Signaling.

[10] (2020). Safety Requirements for Electric Vehicles.

[11] (2020). Safety Requirements for Power Batteries for Electric Vehicles.

[12] Gabbar H.A., Othman A.M., Abdussami M.R. (2021). Review of Battery Management Systems (BMS) Development and Industrial Standards. Technologies.

[13] Cheng K.W.E., Divakar B.P., Wu H., Ding K., Ho H.F. (2011). Battery-Management System (BMS) and SOC Development for Electrical Vehicles. IEEE Trans. Veh. Technol..

[14] Barreras J., Fleischer C., Christensen A., Swiercynski M. (2016). An Advanced HIL Simulation Battery Model for Battery Management System Testing. IEEE Trans. Ind. Appl..

[15] Kharche P., Murali M., Khot G. UDS Impiemetation for ECU I/O Testing. Proceedings of the 2018 3rd IEEE International Conference on Intelligent Transportation Engineering (ICITE).

[16] Wajape M., Elamana N. Study of ISO 14229-1 and ISO 15765-3 and implementation in EMS ECU for EEPROM for UDS application. Proceedings of the IEEE International Conference on Vehicular Electronics & Safety.

[17] Shao L. (2022). Research on the Insulation Performance Monitoring Method of Electric Vehicle Power Battery Pack. Master’s Thesis.

[18] (2017). State Administration of Market Supervision and Administration and National Standardization Administration. Functional Safety Requirements and Test Methods for Battery Management System for Electric Vehicles.

[19] Wang Z. Research on on-line monitoring method of insulation state of electric vehicle high voltage system. Proceedings of the 2020 IEEE International Conference on High Voltage Engineering and Application (ICHVE).

[20] Tzelepis D., Psaras V., Tsotsopoulou E., Mirsaeidi S. (2020). Voltage and current measuring technologies for high voltage direct current supergrids: A technology review identifying the options for protection, fault location and automation applications. IEEE Access.

[21] Lu P. (2021). Crash Safety Study of Pure Electric City Bus. Master’s Thesis.

[22] Jia W., Li C., Qing S. (2021). Comparative study of test standards for battery systems exported to the EU for electric vehicles. Power Technol..

[23] Xia Z., Chao J., Peng Z., Jing Q., Lin L., Hai M. (2019). Review of safety testing standards and specifications for power lithium batteries at home and abroad. Energy Storage Sci. Technol..

[24] Xiong R., Sun W., Yu Q., Sun F. (2020). Research progress, challenges and prospects of fault diagnosis on battery system of electric vehicles. Appl. Energy.

[25] Quan X. (1994). Introduction to standard commands for programmable instruments (SCPI). Comput. Autom. Meas. Control.

[26] Peiran D., Aixue X. (2010). Remote testing system based on general-purpose instruments. Ship Electron. Eng..

[27] Bo L., Jian Z., Yu S., Hai W. (2008). Research and implementation of Linux-based SCPI command interpreter. Comput. Meas. Control.

